# Current knowledge on the prevalence and detection techniques of *Clonorchis sinensis* in China

**DOI:** 10.3389/fvets.2025.1618633

**Published:** 2025-08-13

**Authors:** Jianming Zhang, Jian Cao, Liang Cao, Chao Zhang, Liyuan Gong, Xiaohong Kang

**Affiliations:** Jiuquan Vocational and Technical University, Jiuquan, China

**Keywords:** *Clonorchis sinensis*, prevalence, different hosts, detection approaches, China

## Abstract

**Background:**

*Clonorchis sinensis* is one of the most significant zoonotic food-borne parasites, particularly prevalent in China. The adult form of *C. sinensis* typically inhabits in the hepatic and biliary tracts of various mammals, including humans, and is considered a major contributor to the incidence of cholangiocarcinoma. The life cycle of *C. sinensis* is complex, understanding its prevalence among different host species is essential for developing prevention and management measures. Furthermore, the clinical manifestations of clonorchiasis are often overlooked or misdiagnosed. Therefore, the development of early detection methodologies will facilitate timely treatment and reduce associated complications.

**Methods:**

The current review was conducted in accordance with the guidelines of Preferred Reporting Items for Systematic Reviews and Meta-Analyses. A comprehensive search for relevant articles was performed across PubMed, Web of Science, Google Scholar, China National Knowledge Infrastructure, Wanfang, and Wipro databases. The selected studies investigated the prevalence of *C. sinensis* among various host species in China, and developed the corresponding diagnostic approaches.

**Results:**

Various diagnostic approaches have been established for detecting *C. sinensis.* These methods encompass direct identification, molecular techniques, serological assays, and imaging modalities, each of which targets distinct aspects such as morphological characteristics, DNA sequences, specific antibodies, and the visual representation of *C. sinensis.* The prevalence characteristics of *C. sinensis* across different host species have been examined in China, with the majority of studies concentrating on the second intermediate hosts, specifically fishes, and the definitive hosts, namely humans. The overall prevalence of *C. sinensis* among fish species was found to be 18.36% (4,892/26,646, 95% CI 17.90–18.82), with notably high detection rates observed in the provinces of Heilongjiang, Qinghai, Liaoning, and Shandong. In Chinese human population, the average detection rate of this pathogen was recorded at 3.98% (60,306/1,513,994, 95% CI 3.95–4.01), with the highest rates occurring in the provinces of Jiangxi, Hunan, Guangdong, and Heilongjiang Provinces.

**Conclusion:**

This review emphasized on the widespread prevalence of *C. sinensis* among different host species in China, and summarized the existing diagnosis methods developed in China. These findings will establish a foundational framework for the prevention, control, and potential eradication of clonorchiasis in China.

## 1 Introduction

*Clonorchis sinensis,* initially classified as *Distoma sinensis* in 1875, was first identified in human hosts and is recognized as one of the most significant food-borne parasites, demonstrating widespread prevalence in specific regions of China (1). According to the third national survey on important human parasitic diseases conducted between 2016 and 2021 in China. Approximately 9.46 million (95% BCl, 8.22 million–10.88 million) individuals were estimated to be infected with *C. sinensis* (2). This disease has been prevalent in nineteen provinces and regions of China, with particularly high infection rates reported in Guangxi, Heilongjiang, Guangdong, and Jilin Provinces (2).

**Figure 1:**
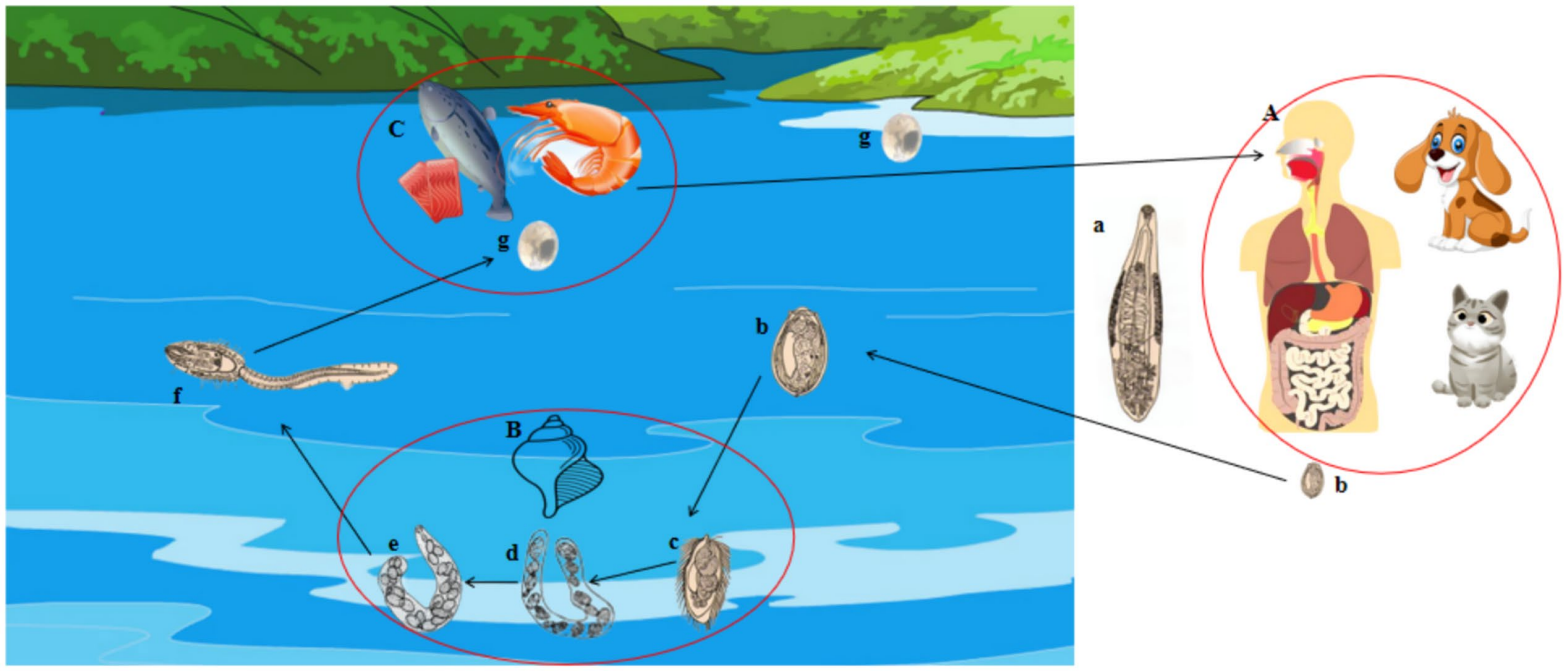
The life cycle of the adult *C. sinensis* is characterized by several distinct stages. These stages include: **(a)** the *C. sinensis* adult; **(b)** the eggs; **(c)** sporocysts, **(d)** sporocysts; **(e)** rediae; **(f)** cercariae; **(g)** metacercariae; **(A)** the definitive hosts for this organism include human beings, dogs, cats, pigs, and other species; **(B)** the first intermediate hosts are represented by various species of freshwater snails; **(C)** the second intermediate hosts consist of freshwater fish and shrimp species.

*C. sinensis* has a multifaceted life cycle characterized by seven developmental stage: eggs, miracidium, sporocyst, redia, cercaria, metacercaria, and adult. Successful completion of its life cycle requires three different types of hosts (Figure 1). In brief, the eggs of *C. sinensis*, excreted in the feces of the definitive host, are released into aquatic environments and ingested by the first intermediate host, freshwater snail. Within the snail’s gastrointestinal tract, the eggs hatch into micracidia, and subsequently develop into sporocysts, rediae, and cercariae (1). The cercariae are then released into the water and infect the second intermediate hosts, including freshwater fish and freshwater shrimp. Inside these hosts, cercariae encyst in muscle tissue and develop into the metacercariae (3, 4). Definitive mammalian hosts (such as humans, cats, and dogs) acquire infection by consuming the raw or undercooked fish or shrimp containing metacercariae, leading to the development of adult worms in the hepatobiliary system (5). Also, infection with *C. sinensis* can lead to severe hepatic and gallbladder damage, resulting in conditions such as pyogenic cholangitis, choleithiasis, and even cholangiocarcinoma (6).

In recent years, changing dietary preferences have led to the increased consumption of raw or semi-raw fish “yusheng” and “drunken shrimp” in specific regions of China (7, 8). These dietary changes may contribute to a higher incidence of *C. sinensis* infections among human populations in China (4). Timely diagnosis is essential for detecting the presence of *C. sinensis*, developing effective prevention strategies, and assessing its prevalence across various host species. This review provides updated information on *C. sinensis* in China, including its diagnostic methods and prevalence among susceptible hosts, which is critical for the prevention and control of this disease.

## 2 Methods

### 2.1 Study protocol

This review was performed in accordance with the guidelines established by the Preferred Reporting Items for Systematic Reviews and Meta-Analyses (PRISMA) (9). The objective was to identify related literature concerning the prevalence of *C. sinensis* across various host species, as well as the advancement of diagnostic methodologies for the detection of this pathogen within China.

### 2.2 Searching strategies and information sources

A comprehensive search was conducted across six databases, namely PubMed, Web of Science, Google Scholar, China National Knowledge Infrastructure, Wanfang, and Wipro, to systematically retrieve relevant articles.

In this review, we intend to synthesize the prevalent characteristics of *C. sinensis* across different host species, with a particular focus on humans and fish, in recent years. Three national surveys have been conducted to investigate the prevalence of significant human parasites, including clonorchiasis, in China during the periods of 1988–1992, 2001–2004, and 2016–2020. Additionally, an analysis of the prevalent characteristics of *C. sinensis* in China from 2000 to 2016 has been undertaken (10). Consequently, this section is confined to research published from 2010 to April 30, 2025 in both Chinese and English databases. The diagnostic methodologies for the identification of *C. sinensis* developed in China have been systematically summarized, encompassing literature published from the time of its initial discovery to the present.

### 2.3 Data synthesis and statistical analysis

Graphs and tables were employed to depict the prevalence of *C. sinensis* across different host species. The pooled prevalence and its corresponding 95% confidence intervals (95% CI) for *C. sinensis* were determined using the epidemiological calculator EpiTools software. Potential risk factors influencing its prevalence in humans, including region, gender, age, occupation, education level, ethnic group, and residential location, were examined through Chi-square test in SPSS version 24.0 (SPSS Inc., Chicago, IL, United States). A *p*-value of less than 0.05 was considered indicative of a statistical difference. In addition, a correlation analysis was conducted using GraphPad. Prism version 9.0 software (GraphPad Software Inc., CA, United States) to assess the concordance between the prevalence of *C. sinensis* in both fish and human populations.

## 3 Results

### 3.1 Current diagnostic methods for detecting *Clonorchis sinensis*

The prevalence of *C. sinensis* poses a significant threat to public health in China, thus early diagnosis is essential for mitigating the impact of the disease and reducing treatment costs for affected individuals. In recent years, a range of diagnostic techniques has been developed for detecting *C. sinensis* in clinical samples, which can be categorized into four primary types: morphological identification that involves the detection of eggs, encysted metacercariae and adult form of *C. sinensis*; molecular methods that target nucleic acids, serological technologies that identify specific antibodies, and the imaging modalities for the visualization of *C. sinensis*. This section presents an overview of these diagnostic methods developed in China, their respective characteristics are summarized in Table 1.

**Table 1:** The pooled prevalence of *C. sinensis* across various fish species in different provinces/regions in China.

Methods	Targets	Operation time	Detection limit	Experimental costs	Main characteristics	Reference
Kato-Katz method	Eggs	45 ~ 75 min	-	*	The Kato-Katz method has been extensively employed to assess the prevalence of *C. sinensis* in clinical samples, owing to its cost-effectiveness and superior sensitivity compared with other direct pathogen detection methods.	(12)
Imaging technologies	The damage and specific morbidity	-	Low sensitivity	****	These imaging technologies, such as cholangiography, exhibit lower values compared with direction pathogen detection techniques.	(16–18)
PCR	Cox1 or ITS genes	1.5 ~ 2.0 h	-	*	PCR methods exhibit superior sensitivity in comparison to direct pathogen detection and imaging techniques, while which require significant time, specialized personnel, and complex equipment.	(20–22)
Multiplex PCR	-	1.5 ~ 2.0 h	10,000 copies/μL	*	In addition to traditional PCR techniques, multiplex PCR methods are capable of concurrently identifying multiple pathogens, including *Clonorchis sinensis*.	(23)
			1 ng/μL	*		(24)
Real-time PCR	-	~60 min	0.1 pg./μL	**	Real-time PCR methods demonstrated higher sensitivity in comparison with the Kato-Katz and conventional PCR methods, but which require expensive equipment.	(26)
			9.97 copies/μL	**		(27)
LAMP	Unit R2 gene	40 min	10 fg/μL	***	The advantages of LAMP include high sensitivity and specificity, no requirement of expensive equipment and complex procedures; while these methods require multiple primers, which may result in high false positive rate.	(31)
	ITS-2 gene	60 min	10 fg/μL	***		(32)
	Cox1 gene	< 40 min	10^−8^ ng/μL	***		(33)
RAA	18S rRNA gene	<10 min	3 pg./μL	***	The advantages of RAA include simple and fast operation with high sensitivity, while this method is characterized by high cost and high false positive rate.	(34)
	-	<20 min	1 *C. sinensis*	***		
RPA	Cox1 gene	15 min	2.75 ng/μL	***	The benefits and drawbacks are comparable to those associated with RAA techniques.	(37)
	Exo gene	20 min	10 fg/μL for *C. sinensis* genome and 100 copies/μL for the plasmid	***		(38)
RPA combined with LFD	Exo gene	20 min	10 fg/μL for the DNA genome	Higher than RPA (****)	The benefits and drawbacks are comparable to those associated with RAA techniques. Furthermore, the detection outcomes can be observed visually.	(39)
PCR-CRISPR/Cas12	ITS gene	1.5 ~ 2.0 h	0.1 copies/μL of the recombinant plasmid	***	The sensitivity of the method has been significantly enhanced relative to traditional PCR techniques, and the results can be visualized without the need for a gel electrophoresis system.	(40)
RPA-CRISPR/Cas12a	Cox1 gene	Within 60 min	0.8 metacercariae per gram of fish sample	$2.55 per sample (***)	The sensitivity of this method has been greatly improved in comparison with the conventional RPA method, while which is characterized by higher cost.	(41)
RPA-CRISPR/Cas12a combined with LFD	Cox1 gene	Within 60 min	5 metacercariae per gram of fish sample	$4.85 per sample (****)	The detection outcomes can be observed visually, while this methods require higher cost compared with the RPA/CRISPR-Cas12 method	(41)
Indirect ELISA	Enolase protein	-	-	**	These methods specifically recognize the antibodies against *C. sinensis* in canines, which demonstrate higher sensitivity compared with the enhanced Kato-Katz method.	(44)
Double antibody sandwich ELISA	Excretory/secretary antigen	-	The antibodies of 0.014 μg/mL	**		(45)

The symbols “*”, “**”, “***”, and “****” denote the relative costs of the corresponding detection methods, ranging from low to very high.

#### 3.1.1 Traditional detection methods

##### 3.1.1.1 Direct examination

The detection of *C. sinensis* in fecal samples is considered the “gold standard” for identifying eggs. This diagnostic process incorporates various techniques, including the saturated saltwater flotation method (11), water washing precipitation method (12), and Kato-Katz method (13). Among them, the Kato-Katz method stands is particularly notable for its enhanced sensitivity compared to other diagnostic techniques, which is frequently used to assess the prevalence of *C. sinensis* in stool samples among humans and other definitive hosts (13–15).

##### 3.1.1.2 Imaging techniques

Imaging techniques, including ultrasonography, computed tomography (CT), magnetic resonance imaging (MRI), and cholangiography, have been extensively utilized to detect the damage and specific pathologies associated with particular specific organs and tissues. This application aids in assessing the potential impacts of *C. sinensis* and other parasitic infections (16).

Among these techniques, cholangiography exhibits a higher sensitivity in detecting the presence of *C. sinensis* in comparison to other imaging methods (17, 18). The sensitivity and specificity of these imaging technologies are considerably influenced by disease severity, demonstrating lower values when compared to other alternative direct pathogen detection methods. Furthermore, the majority these applications have been primarily focused on the identification of pathogens in human subjects.

#### 3.1.2 Molecular detection approaches

##### 3.1.2.1 Polymerase chain reaction (PCR)

In recent years, conventional PCR techniques have been widely employed for molecular diagnosis of *C. sinensis*, demonstrating high sensitivity and specificity (19–22). These established PCR methods primarily target conserved genomic regions of *C. sinensis*, such as the internal transcribed spacer (ITS) and the mitochondrial cytochrome c oxidase subunit 1 (cox1) genes (20). Given the concurrent presence of multiple trematode species in fish, various multiplex PCR approaches have been developed to enable the simultaneous detection of *C. sinensis* alongside other species (23, 24). For example, Gao et al. developed a multiplex PCR method for the identification of *C. sinensis*, *Metorchis orientalis*, and *Holostephanus dubinini*, with a detection limit of 10,000 copies/μL for all three trematode species (23). Similarly, Li et al. introduced a novel multiplex PCR technique that could concurrently detect the presence of *C. sinensis*, *M. orientalis*, *Metagonimus yokogawai*, *Echinochasmus japonicus* and *Echinostoma hortense*, achieving a detection limit of 1 ng/μL for *C. sinensis* (24).

##### 3.1.2.2 Real-time PCR

Real-time PCR techniques have been widely employed for early diagnosis of various pathogens in clinical samples, primarily owing to their enhanced sensitivity and accuracy in comparison to traditional PCR methodologies (25). Qiao et al. developed a TaqMan-based real-time PCR method for the detection of *C. sinensis* in gallbladder stone samples, achieving a detection limit of 0.1 pg./μL (26). More recent, a novel TaqMan-based real-time PCR assay has been established for the identification of *C. sinensis* eggs in fecal samples, which reached a detection limit of 9.97 copies/μL and exhibited superior sensitivity relative to the modified Kato-Katz method (27). In a related vein, Cai et al. introduced an assay that integrates real-time PCR method with high-resolution melting analysis, facilitating the differentiation between *C. sinensis* and *O. viverrini*, with a detection limit of 5 eggs per gram, 1 metacercaria, or less than 1 pg. of the purified DNA genome of *C. sinensis* (28).

##### 3.1.2.3 Loop-mediated isothermal amplification (LAMP)

LAMP is a nucleic acid amplification technique that operates under constant temperature conditions (29). LAMP was initially invented in 2000, it has since been widely applied for the molecular detection of various pathogens (30). In a notable study, Shi et al. introduced a novel LAMP method that specifically targeted the ITS-2 gene of *C. sinensis*, achieving a detection limit of 10 pg./μL (31). In a similar vein, Sun and colleagues used the Primer Explorer 5.0 software to design three pairs of primers directed at the Unit R2 gene of *C. sinensis*, thereby establishing an effective LAMP technique (32). This method exhibited a detection limit of 10 fg/μL, demonstrating a sensitivity that was 100 times greater than that of conventional PCR, with results obtainable within a 40-min timeframe (32).

##### 3.1.2.4 Recombinase aided isothermal amplification (RAA)

The RAA technique, akin to the LAMP method, is an isothermal rapid amplification technology used for detecting nucleic acids. This approach is characterized by its ability to maintain constant temperatures, typically between 37°C and 42°C, and offering high specificity, efficiency, and straightforward operational protocols (33). Zhang et al. (34) utilized a conserved region of the 18S rRNA sequence from *C. sinensis* to design a pair of primers for developing a RAA method, which demonstrating a detection limit of 10 copies/μL of the recombinant plasmid and 3 pg./μL of the *C. sinensis* genome. Notably, the positive results obtained through this innovative approach could be monitored within 10 min, making it suitable for the detection of *C. sinensis* in fecal and fish samples (34). Chen et al. (35) used a commercially available RAA detection kit to assess its effectiveness in identifying *C. sinensis* in freshwater fish specimens. The findings revealed that this kit was capable of specifically detecting a single *C. sinensis* metacercaria in a 5-gram sample of freshwater fish within 20 min. Furthermore, the detection rate of *C. sinensis* using the RAA method was found to be significantly superior to that achieved through the direct compression method (35).

##### 3.1.2.5 Recombinase polymerase amplification (RPA)

RPA is a modern isothermal amplification technique that was first introduced in 2006. RPA has gained recognition as a viable alternative to LAMP and RAA methods, due to its numerous advantageous characteristics, such as cost-effectiveness, high sensitivity, rapid processing times, ease of use, and the minimal equipment requirements (36). Zhang et al. (37) employed the conserved region of the cox1 gene from *C. sinensis* to establish a rapid RPA technique, which can be conducted within 15 min at a constant temperature of 35°C. This innovative method demonstrated a detection limit of 2.75 ng/μL, the results can be visually interpreted without the necessity for specialized equipment. Similarly, Zhang HY developed an RPA method targeting the Exo gene of *C. Sinensis*, which can be performed at a constant temperature of 39°C and completed within 20 min (38). Moreover, this technique demonstrated high specificity in detecting the nucleic acids of *C. sinensis* across various developmental stages, with a detection limit of 10 fg/μL for the DNA genome and 100 copies/μL for the plasmid (38).

In a recent investigation, Ma et al. (39) devised a rapid and visually interpretable method for identifying *C. Sinensis,* utilizing a combination of RPA and lateral flow dipstick technology. The sensitivity of this innovative technique was 10 fg/μL for the DNA genome, with the ability to detect fewer than one purified egg or metacercariae. Furthermore, this technique was performed at 39°C within 20 min, indicating its potential as an alternative tool for the identification of *C. sinensis* in clinical specimens (39).

##### 3.1.2.6 CRISPR/Cas12 detection system

The clustered regularly interspaced short palindromic repeats (CRISPR) system was identified within the genomes of bacteria and archaea, serving as a “gene weapon” to defend against the intrusion of foreign genetic material (40). Presently, CRISPR/CRISPR-associated protein (Cas) systems are extensively used across diverse domains and are classified into several types based on the genetic variability of Cas gene (29). Among these systems, the CRISPR/Cas12a system has emerged as a promising approach for nucleic acid detection (27). Usually, the sensitivity and convenience of CRISPR/Cas12a system can be significantly improved when combined with the isothermal amplification techniques, such as RPA (41) and LAMP (42).

Xu et al. developed a novel PCR-CRISPR/Cas12 technology for the rapid detection of *C. sinensis* (43). In this approach, the conserved region of the ITS gene from *C. sinensis* was amplified using PCR, the resultant PCR products were subsequently introduced into the CRISPR/Cas12a system, enabling the evaluation of detection outcomes based on fluorescence signal intensity (43). The detection limit of this methodology was 0.1\u00B0copies/μL of the recombinant plasmid; however, the operational time was significantly longer compared to other isothermal amplification techniques (40). To mitigate this drawback, an alternative research group proposed a portable detection platform using RPA-CRISPR/Cas12a with dual readout capabilities, specifically targeting the mitochondrial cox1 gene of *C. sinensis* (41). This innovative method can be completed within 60 min and has a detection limit of approximately 0.8 metacercariae per gram of fish sample, with an estimated cost of 2.55 dollars (41). When this method is combined with a lateral flow assay, the detection limit increases to 5 metacercariae per gram of fish sample, resulting in a total cost of 4.85 dollars (41).

#### 3.1.3 Serological approaches for the detection of *Clonorchis sinensis*

Currently, there are no commercially available vaccines for *C. sinensis* infection in China, thus serological detection methods can be applied to distinguish between infections caused by *C. sinensis* and those uninfected individuals. In recent years, a variety of serological techniques, primarily based on ELISA methodologies, have been developed to identify the specific antibodies against *C. sinensis* (44, 45). The identification of soluble antigens with high sensitivity and specificity is essential for the enhancement of ELISA methodologies.

Numerous studies have assessed various antigens suitable for the development of ELISA methods. For example, Pan et al. utilized the enolase protein from *C. sinensis* to create an indirect ELISA, which specifically detected antibodies against *C. sinensis* in canine subjects (44). Furthermore, the specificity of this ELISA method was markedly superior to that of the enhanced Kato-Katz method, yielding detection rates of 1.13% (4/353) and 0.28% (1/353), respectively (44). Similarly, Zhu et al. used the excretory/secretory antigen (EAS) of *C. sinensis* to establish a double antibody sandwich ELISA method (45). This technique demonstrated high sensitivity and specificity, enabling the specific identification of antibodies against *C. sinensis* with a detection limit of 0.014 μg/mL (45).

To improve the detection efficiency and optimize the testing process, Ma et al. developed a point-of-care testing assay for detecting *C. sinensis* using a Eu-(III) nanoparticles (EuNPs)-tandem repeat sequence 1 (CSTR1) fluorescent probe-based immunoassay (46). In this methodology, EuNPs functioned as signaling probes, which were conjugated with the CSTR1 antigen to selectively binding antibodies specific to *C. sinensis*. This complex was then analyzed on a test line that emitted a fluorescent signal upon exposure to ultraviolet light (46). Overall, this innovative approach facilitated the specific detection of antibodies against *C. sinensis*, achieving a detection limit of serum samples corresponding to 24 eggs per gram in fecal examinations, and could be completed within a 10-min timeframe, thereby enhancing the feasibility of point-of-care detection for *C. sinensis* (46).

### 3.2 Prevalence of *Clonorchis sinensis* among various hosts in China

#### 3.2.1 Literature search

**Figure 2:**
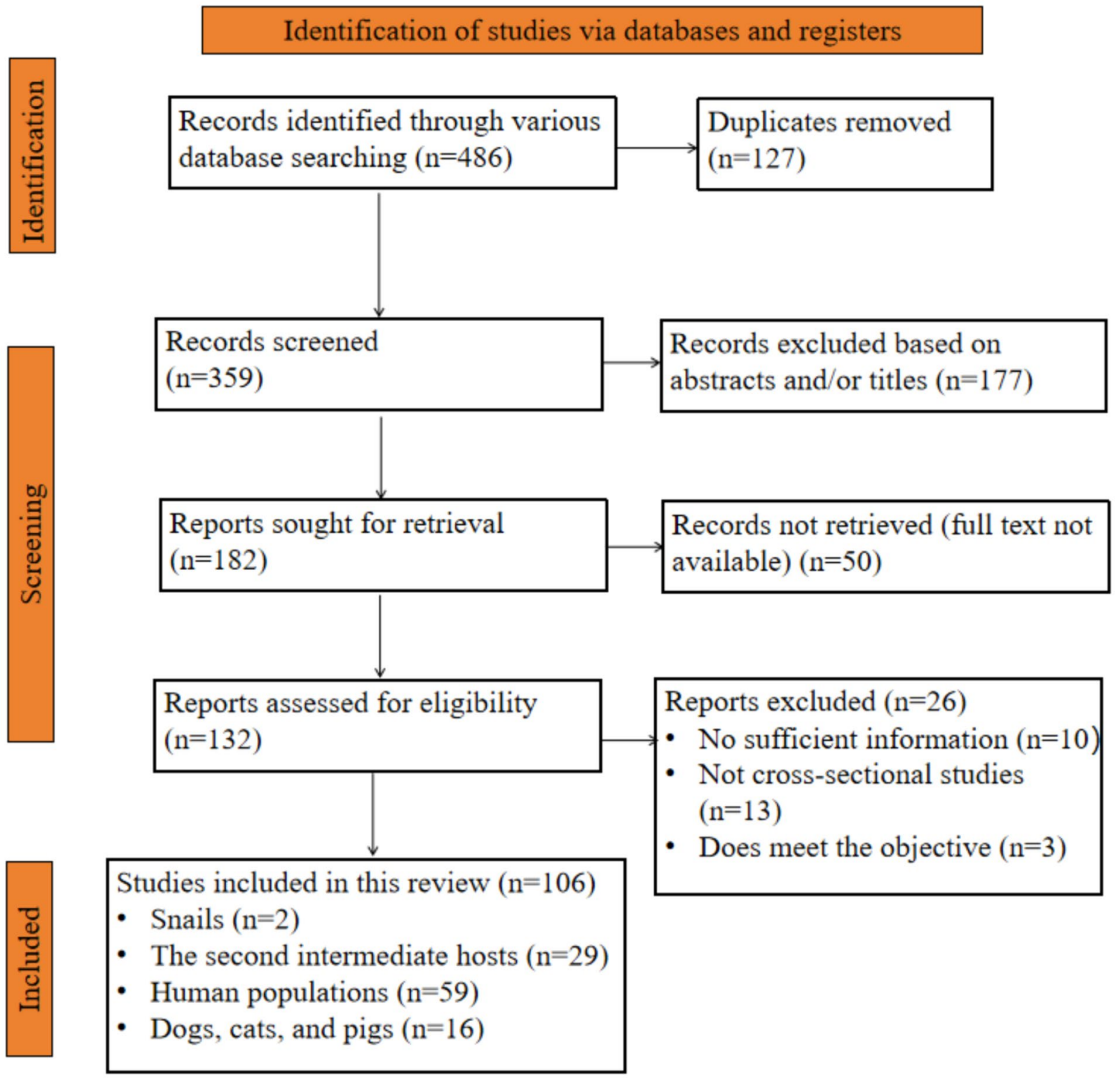
Flow chart of the selected studies that examine the prevalence of *C. sinensis* across various host species in China.

As illustrated in the PRISM 2020 flow chart (Figure 2), a comprehensive search across six databases yielded a total of 486 articles examining the prevalence of *C. sinensis* among different host species in China from 2010 to April 30, 2025. Following the removal of 127 duplicate entries, 359 articles remained. Subsequently, 177 articles were eliminated based on the title and abstract screening, and an additional 50 articles were not available in full text, leading to a total of 132 articles being evaluated for eligibility. However, 26 reports were excluded for several reasons, resulting in the inclusion of 106 studies in this analysis.

#### 3.2.2 The prevalence of *Clonorchis sinensis* among the first intermediate hosts

Snails serve as the primary intermediate host for *C. sinensis*. Numerous fresh-water snail species are distributed across different regions of China, and more than eight species have been reported to be infected with *C. sinensis* (1). However, the prevalence of *C. sinensis* among these snails has often been neglected in recent years. Chen et al. developed a LAMP method to detect *C. sinensis* in freshwater snails and reported an average detection rate of 19.66% (92/468) (47). The prevalence in *Bithynia fuchsianus*, *Parafossarulus striatulus*, and *Alocinma longicornis* were 33.33% (2/6), 19.46% (87/447), and 20.0% (3/15), respectively (47). In a previous research, 73 (1.15%, 73/630) snails tested positive for *C. sinensis* eggs in the Zhujiang Delta of Guangdong Province (48). The detection rates among *P. striatulus*, *Parafossarulus sinensis*, *B. fuchsianus,* and *A. longicornis* were 20.0% (41/204), 11.8% (13/110), 12.3% (12/97), and 8.2% (7/85), respectively (48).

#### 3.2.3 The prevalence of *Clonorchis sinensis* among the second intermediate hosts

**Figure 3:**
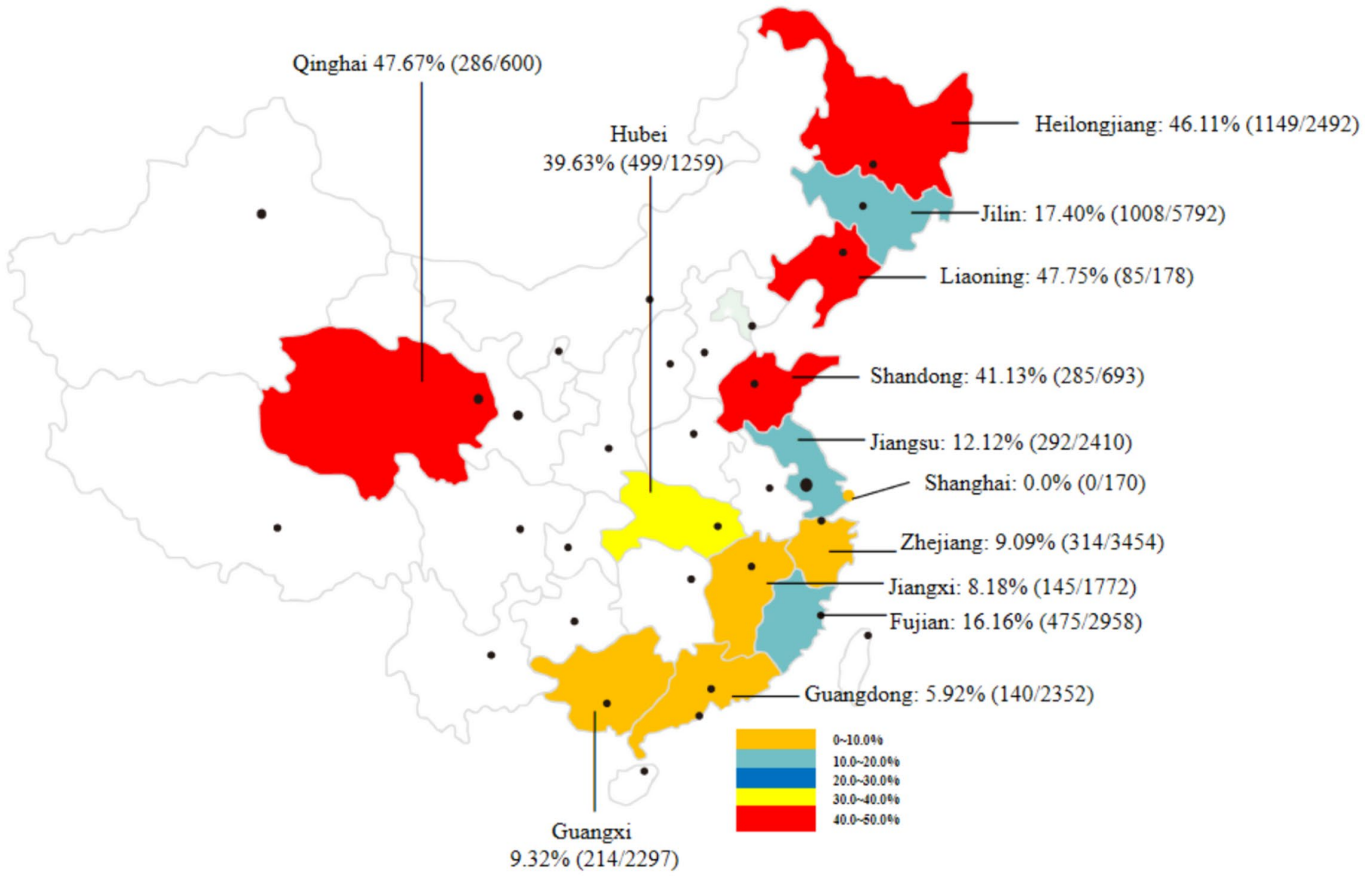
An overview of the prevalence of *C. sinensis* in various second intermediate hosts from different provinces and regions in China from 2010 to April 30, 2025.

To assess the prevalence of *C. sinensis* in second intermediate hosts (mainly referred to freshwater fish) in China in recent years, we reviewed 29 representative studies (Supplementary Table 1) conducted from 2010 to to April 30, 2025 covering 13 provinces and cities in China. A total of 26,646 freshwater fish samples were included, among which 4,892 were found to be positive for *C. sinensis* metacercariae, resulting in an overall infection rate of 18.36% (95% CI 17.90–18.82). As shown in Figure 3, the prevalence of *C. sinensis* varied widely by region ranging from 0.0% in Shanghai City to 47.75% in Liaoning Province. Notably, four provinces, Heilongjiang, Qinghai, Liaoning, and Shandong, had infection rates exceeding 40%, while five provinces reported ranged between 0.0 to 10.0%.

As presented in Table 2, the prevalence of *C. sinensis* has been investigated in 30 fish species across China. Among these species, *Saurogobio dabryi* and *Abbottina rivularis* exhibited the highest detection rates, at 60.53% (172/284) and 59.73% (89/149), respectively. Several freshwater fish species, include *Pseudorasbora parva*, *Carassius auratus*, *Cyprinus rubrofuscus*, *Hemiculter leucisculus*, *Hypophthalmichthys nobilis*, and *Rhodeus sericeus*, have been frequently studied for the prevalence of *C. sinensis* in China. The average detection rates of these freshwater fishes were 25.68% (4,129/16,073). In addition to freshwater fish, Yu et al. reported an average detection rate of 2.96% (34/1,149) for *C. sinensis* in samples of *Macrobrachium nipponense* collected from Zhejiang Province, China (49).

**Table 2:** The pooled prevalence of *C. sinensis* among human beings in China.

Factor	Category	No. of studies	No. of tested samples	No. of positive samples	% (95% CI)
Provinces/regions	Fujian	3	2,985	475	16.06 (14.74–17.38)
	Zhejiang	4	3,545	314	9.09 (8.14–10.03)
	Jiangsu	3	2,410	292	12.12 (10.82–13.42)
	Qinghai	1	600	286	47.67 (43.67–51.67)
	Guangxi	2	2,297	214	9.32 (8.13–10.51)
	Heilongjiang	3	2,492	1,149	46.11 (44.15–48.06)
	Hubei	2	1,259	499	39.63 (36.92–42.33)
	Jilin	3	5,792	1,008	17.40 (16.42–18.38)
	Jiangxi	1	1772	145	8.12 (6.85–9.39)
	Shanghai	1	271	0	0.0
	Liaoning	1	178	85	47.75 (40.41–55.09)
	Guangdong	4	2,352	140	5.92 (5.72–6.12)
	Shandong	1	693	285	41.13 (37.47–44.79)
	Fujian	3	2,985	475	16.06 (14.74–17.38)
Fish species	*Pseudorasbora parva*	22	8,078	3,140	38.87 (37.81–39.93)
	*Carassius auratus*	21	3,597	330	9.17 (8.22–10.11)
	*Cyprinus rubrofuscus*	13	620	27	4.35 (2.74–5.96)
	*Hemiculter leucisculus*	12	1755	270	15.38 (13.69–17.07)
	*Hypophthalmichthys nobilis*	10	602	43	7.14 (5.08–9.20)
	*Rhodeus sericeus*	10	1,421	319	22.45 (20.28–24.62)
	*Ctenopharyngodon idella*	6	527	20	3.76 (2.13–5.38)
	*Misgurnus anguillicaudatus*	6	1,356	11	0.81 (0.03–1.29)
	*Hypophthalmichthys molitrix*	5	249	13	5.22 (2.46–7.98)
	*Pelteobagrus fulvidraco*	5	147	3	2.04 (0–4.31)
	*Saurogobio dabryi*	5	284	172	60.53 (54.84–66.21)
	*Phoxinus lagowskii*	5	251	113	45.02 (38.86–51.57)
	*Parabramis pekinensis*	4	376	53	14.09 (10.57–17.61)
	*Oreochromis. spp*	4	538	23	4.28 (2.57–2.60)
	*Ctenopharyngodon idella*	4	520	50	9.62 (7.09–12.15)
	*Leptoscopus maculatus*	3	114	25	21.93 (14.33–29.53)
	*Monopterus albus*	3	756	10	1.32 (0.51–2.13)
	*Cirrhinus molitorella*	3	196	16	8.16 (4.33–11.99)
	*Ctenogobius giurinus*	3	665	47	7.07 (5.12–9.02)
	*Odontobutis obscura*	3	260	34	13.08 (8.98–17.18)
	*Micropterus. spp*	2	51	1	1.96 (0.13–5.76)
	*Spinibarbus denticulatus*	2	84	3	3.57 (1.05–6.54)
	*Macrobrachium nipponense*	2	2,298	68	2.96 (2.27–3.65)
	*Epinephelus. spp*	2	151	5	3.31 (0.46–6.16)
	*Siniperca kneri*	2	67	1	1.49 (0–4.39)
	*Tilapia*	1	31	0	0
	*Astronotus ocellatus*	1	82	10	12.2 (5.12–19.28)
	*Squaliobarbus curriculus*	1	76	5	6.58 (1.00–12.15)
	*Katsuwonus pelamis*	1	10	1	10.0 (0–28.59)
	*Abbottina rivularis*	1	149	89	59.73 (51.86–67.60)
	*Channa argus*	1	11	1	9.09 (0–26.08)

#### 3.2.4 The prevalence of *Clonorchis sinensis* among the definitive hosts in China

##### 3.2.4.1 The prevalence of *Clonorchis sinensis* among humans in China

**Figure 4:**
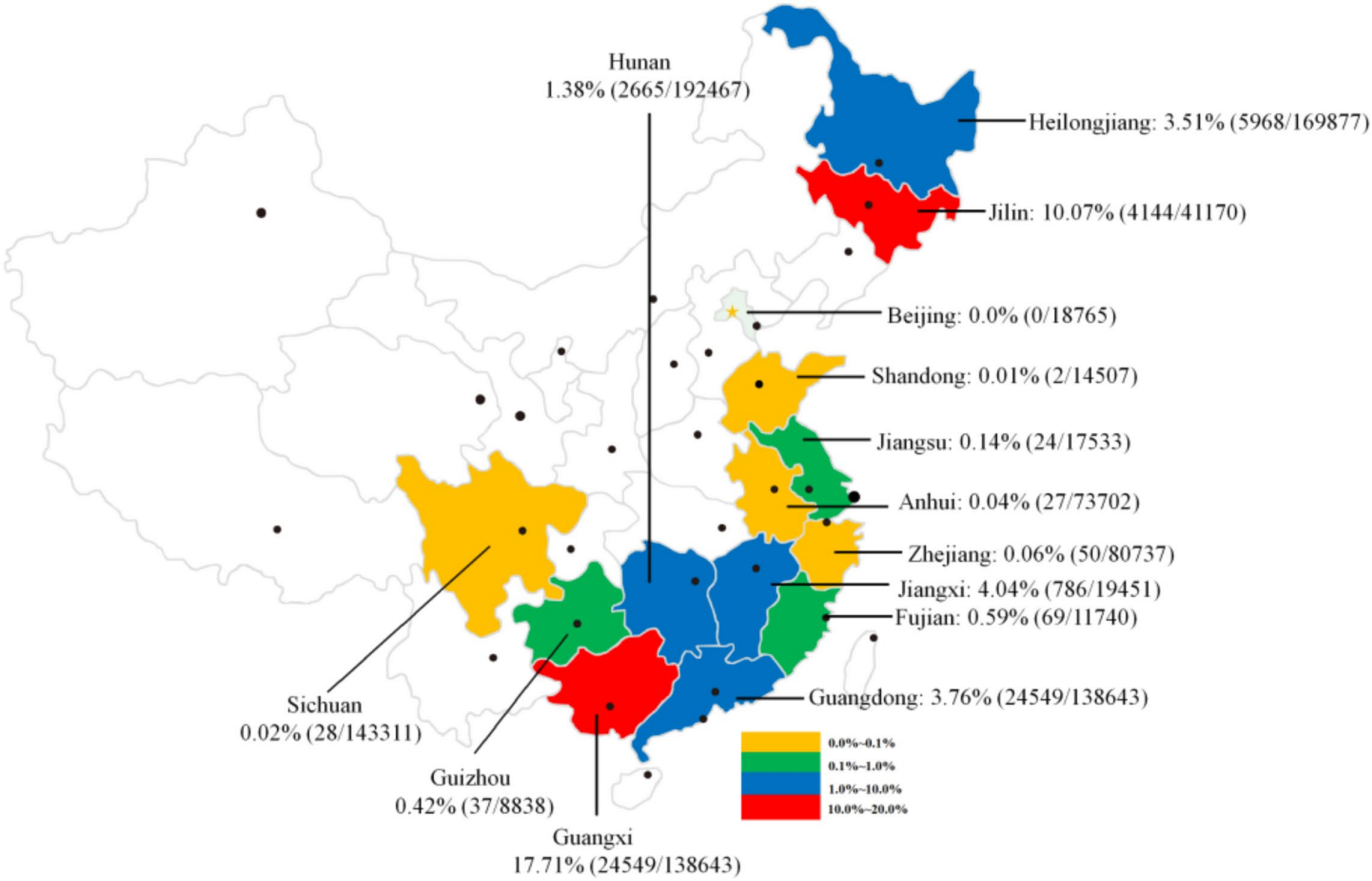
An overview of the prevalence of *C. sinensis* among human populations across various provinces and regions in China, spanning from 2010 to April 30, 2025.

A variety of definitive hosts, including humans, dogs, cats, pigs, and other mammals, are susceptible to *C. sinensis* infection. This review compiled 59 representative studies (Supplementary Table 2) that investigated the prevalence of *C. sinensis* among human population in China from 2010 to April 30, 2025. In total, 1,513,994 samples were analyzed across 14 provinces or regions, with 60,306 samples testing positive for either the eggs or specific antibodies against *C. sinensis*, yielding an overall detection rate of 3.98% (95 CI 3.95–4.01). The detection rates of *C. sinensis* exhibited considerable variation by province, ranging from 0.0% (0/18,765) to 17.71% (24,549/138,643). The highest prevalence was observed in Guangxi (17.71%, 24,549/138,643) and Jilin (10.07%, 4,144/41,170), both exceeding 10.0%. In the provinces of Jiangxi, Hunan, Guangdong, and Heilongjiang, the prevalence rates ranged from 1.0 to 10.0%, while other provinces, including Beijing, Shandong, Jiangsu, Anhui, Zhejiang, Fujian, and Guizhou, reported prevalence rates below 1.0% (Figure 4). A recent report indicated comparable prevalence characteristics of *C. sinensis* infection across various provinces, highlighting a notably high crude prevalence in Guangxi, Heilongjiang, Jilin, and Guangdong Provinces (2).

Next, we conducted an analysis of various potential risk factors associated with the prevalence of *C. sinensis*, including age, gender, occupation, educational attainment, ethnic groups, and residential location. As demonstrated in Table 3, the data indicated a significant gender disparity in infection rates, with males exhibiting a higher prevalence of *C. sinensis* infection (6.70%, 33,741/503,477) compared to females (3.10%, 15,879/511,808). Moreover, an analysis of age-related prevalence indicated that the highest detection rate of *C. sinensis* was observed in individuals aged 30 to 59 years (8.20%, 30,839/376,094), followed by those aged over 60 years (4.99%, 8,650/173,487), while the lowest rate was recorded in individuals under 30 years (1.93%, 4,636/240,048).

**Table 3:** Summary of the detection methods targeting *C. sinensis* and their characteristics in China.

Factor	Category	No. of studies	No. of tested samples	No. of positive samples	% (95% CI)	*p* value
Region	Anhui	1	73,702	27	0.04 (0.03–0.05)	0.101
	Beijing city	4	18,765	0	0.00	-
	Fujian	2	11,740	69	0.59 (0.45–0.73)	<0.05
	Guangdong	10	583,253	21,957	3.76 (3.71–3.81)	<0.05
	Guangxi	17	138,643	24,549	17.71 (17.51–17.91)	<0.05
	Guizhou	1	8,838	37	0.42 (0.29–0.55)	<0.05
	Heilongjiang	5	169,877	5,968	3.51 (3.42–3.60)	<0.05
	Hunan	2	192,467	2,665	1.38 (1.33–1.43)	<0.05
	Jilin	5	41,170	4,144	10.07 (9.78–10.36)	<0.05
	Jiangsu	2	17,533	24	0.14 (0.08–0.20)	<0.05
	Jiangxi	4	19,451	786	4.04 (3.76–4.32)	<0.05
	Shandong	1	14,507	2	0.01 (0.0–0.02)	Reference
	Sichuan	1	143,311	28	0.02 (0.01–0.03)	0.21
	Zhejiang	4	80,737	50	0.06 (0.04–0.08)	<0.05
Gender	Male	46	503,477	33,741	6.70 (6.63–6.77)	<0.05
	Female	46	511,808	15,879	3.10 (3.05–3.15)	Reference
Age	<19	32	178,063	1726	0.10 (0.08–0.11)	Reference
	20 ~ 29	32	61,985	2,910	4.69 (4.52–4.86)	<0.05
	30 ~ 39	32	113,527	8,256	7.27 (7.12–7.42)	<0.05
	40 ~ 49	32	129,151	11,419	8.84 (8.69–8.99)	<0.05
	50 ~ 59	32	133,416	11,164	8.37 (8.22–8.52)	<0.05
	60 ~ 69	32	113,948	6,373	5.59 (5.46–5.72)	<0.05
	>70	32	59,539	2,277	3.82 (3.67–3.97)	<0.05
Occupation	Preschoolers	9	12,753	69	0.54 (0.41–0.67)	Reference
	Students	23	172,254	1,522	0.88 (0.84–0.92)	<0.05
	Workers	19	48,228	2,165	4.49 (4.31–4.67)	<0.05
	Farmers	25	442,408	18,587	4.20 (4.14–4.26)	<0.05
	Cadres or staffs	16	41,955	2,646	6.31 (6.08–6.54)	<0.05
	Medical staffs	10	2022	159	7.86 (6.69–9.03)	<0.05
	Teachers	7	2,126	60	2.82 (2.12–2.52)	<0.05
	Service staffs	14	29,126	988	3.39 (3.18–3.60)	<0.05
	Retiree	10	4,791	52	1.09 (0.80–1.38)	<0.05
Educational attainment	Preschoolers	13	61,270	255	0.42 (0.37–0.47)	Reference
	Illiteracy	26	65,735	1,477	2.25 (2.14–2.36)	<0.05
	Primary school	27	256,126	5,314	2.07 (2.01–2.13)	<0.05
	Middle school	28	224,858	9,370	4.17 (4.09–4.25)	<0.05
	High school or secondary specialized school	26	89,669	4,692	5.23 (5.08–5.38)	<0.05
	University or above	22	42,938	2,513	5.85 (5.63–6.07)	<0.05
Ethnic groups	Han	13	518,438	24,612	4.75 (4.69–4.81)	<0.05
	Zhuang	8	28,688	4,216	14.70 (14.29–15.11)	<0.05
	Man	4	2,404	10	0.42 (0.61–0.68)	<0.05
	Menggu	2	496	36	7.26 (4.98–9.54)	<0.05
	Chinese Korean	3	1,696	166	9.79 (8.38–11.20)	<0.05
	Dong	4	3,193	760	23.80 (22.32–25.28)	<0.05
	Miao	4	2,497	512	20.65 (19.06–22.24)	<0.05
	Buyi	1	845	1	0.12 (0–0.35)	Reference
	Gelao tribe	1	275	0	0.0	-
	Hui	2	210	0	0.0	-
	Yi	1	169	0	0.0	-
	Tujia	1	101	0	0.0	-
	Li	1	75	0	0.0	-
	Yao	5	4,670	480	8.48 (7.68–9.28)	<0.05
Residential location	Rural area	5	50,260	2,612	5.20 (5.01–5.39)	<0.05
	Town and city	5	16,655	648	3.89 (3.60–4.18)	Reference
	Total	59	1,513,994	60,306	3.98%	

The aggregated detection rates of *C. sinensis* by occupation demonstrated that medical personnel and administrative staff had the highest detection rates, recorded at 7.86% (159/2,022) and 6.31% (2,646/41,955), respectively. The prevalence of *C. sinensis* among laborers and agricultural workers was 4.49% (2,165/48,228) and 4.20% (18,587/442,408), respectively. However, students and preschool-aged children exhibited the lowest detection rates, recorded at 0.88% (1,522/172,254) and 0.54% (69/12,753), respectively. In terms of educational attainment, the incidence of *C. sinensis* infection in humans showed a positive correlation with educational levels. Specifically, individuals with a bachelor’s degree or higher had the highest infection rate of infection at 5.85% (2,513/42,038), whereas those with preschool-aged children had the lowest detection rate at 0.42% (255/61,270). Moreover, the detection rate of *C. sinensis* infection among individuals residing in rural areas (5.20%, 2,612/50,260) was significantly higher than those individuals living in urban settings (3.89%, 648/16,655) in China.

##### 3.2.4.2 The prevalence of *Clonorchis sinensis* among other definitive animals in China

**Figure 5:**
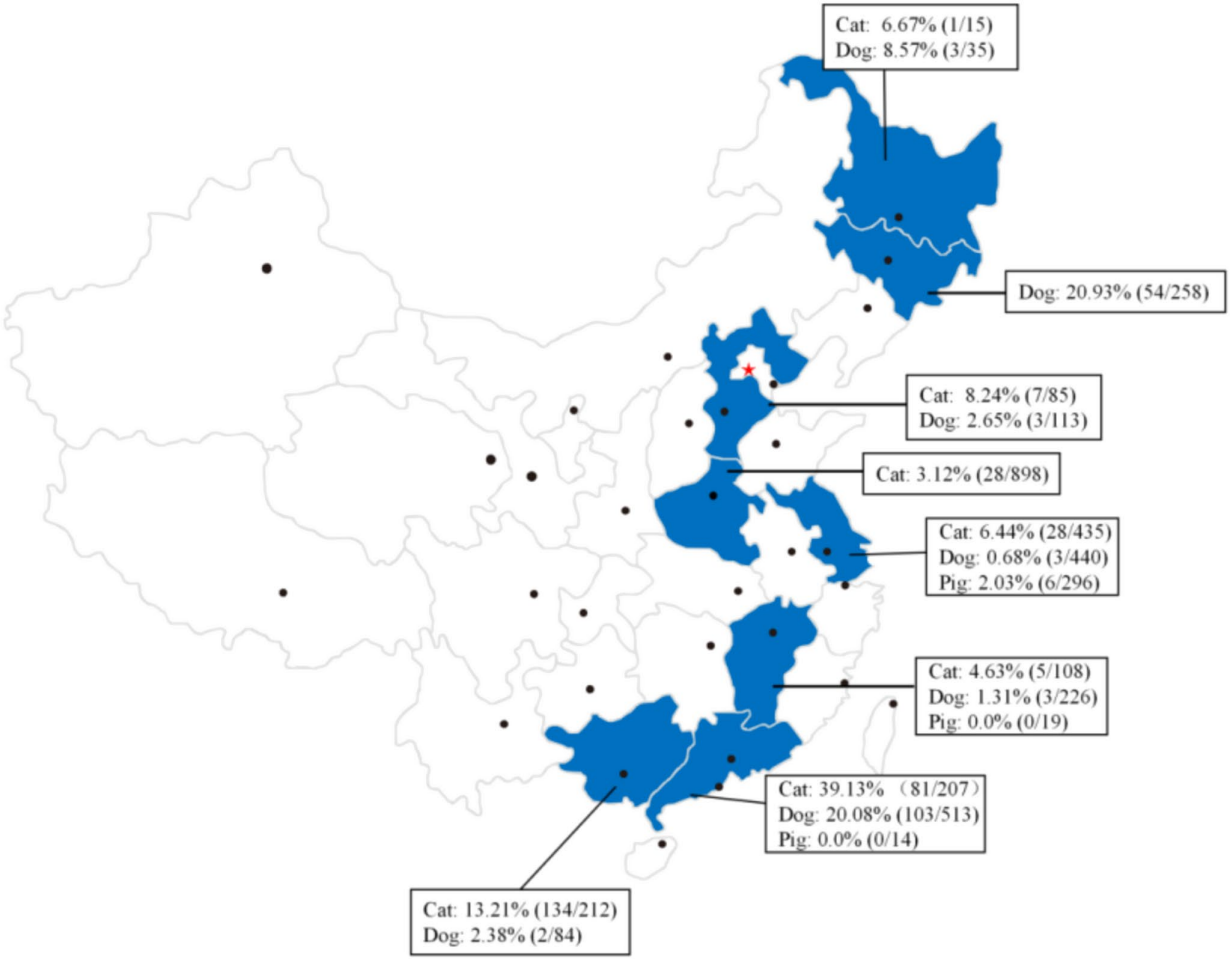
Summary of the prevalence of *C. sinensis* in canine, feline, and pigs populations across various provinces and regions in China.

In recent years, the infection of *C. sinensis* has been documented not only in humans but also in various animal reservoirs including cats (49, 50), dogs (50), pigs (51), rats (52), and forest musk deer (53). However, research has primarily focused on the prevalence of this pathogen in cats, dogs, and pigs in China (54). In detail, the prevalence of *C. sinensis* infection among dogs, cats and pigs have been recorded across eight provinces in China (Figure 5), with the average detection rates of 10.25% (171/1,669) for dogs, 14.50% (284/1,958) for cats, and 1.82% (6/329) for pigs.

## 4 Perspective and conclusion remarks

*Clonorchis sinensis* has been acknowledged as a significant risk factor that considerably affects public health in certain regions of China (55, 56). Additionally, this pathogen is identified a major contributor to hepatobiliary diseases, such as cholelithiasis and cholangiocarcinoma (57, 58). The average medical costs for outpatient and inpatient care related to the treatment of *C. sinensis* infection are 21.72 dollars and 913.80 dollars, respectively (59). This information underscore the considerable economic burden that the widespread occurrence of this parasitic disease places on the public (59). Therefore, it is essential to develop rapid detection approaches and to conduct comprehensive investigations into the prevalence of *C. sinensis* across various host species, as these initiatives are vital for the prevention and control of the disease.

Given the significant public health concerns linked to *C. sinensis* infection, a range of detection methodologies have been developed to evaluate the prevalence of this organism across various host species in China. These methodologies can be primarily classified into four distinct categories: traditional parasitological techniques (e.g., Kato-Katz method), imaging modalities, immunological methods, and molecular techniques. Among these, traditional parasitological methods are frequently used to determine the prevalence of *C. sinensis* infection in different host species. It is noteworthy that the detection rate of *C. sinensis* using molecular methods, such as PCR, is considerably higher than that achieved with the Kato-Katz method (60). Furthermore, similar to the limitations inherent in the Kato-Katz method, ELISA techniques have low specificity and sensitivity, particularly in instances of mild infection intensity (61).

In light of these challenges, a range of molecular techniques has been developed for detecting *C. sinensis*, aimed at overcoming the limitations of serological and traditional parasitological methods. However, the implementation of these molecular techniques in clinical studies assessing the prevalence of *C. sinensis* has been restricted, which may be due to several inherent drawbacks: (1) conventional molecular detection methods, such as PCR and real-time PCR, require complex procedural steps, expensive instrumentation, and considerable time commitments; (2) these innovative visual detection methods, including LAMP (31–33), RPA (37, 38), RAA (34), and the combination of CRISPR/Cas12a with RPA (41), do not demand costly and sophisticated equipment. However, their high sensitivity may lead to an increased occurrence of false-positive results, and the associated high costs of these techniques have further limited their widespread application.

**Figure 6:**
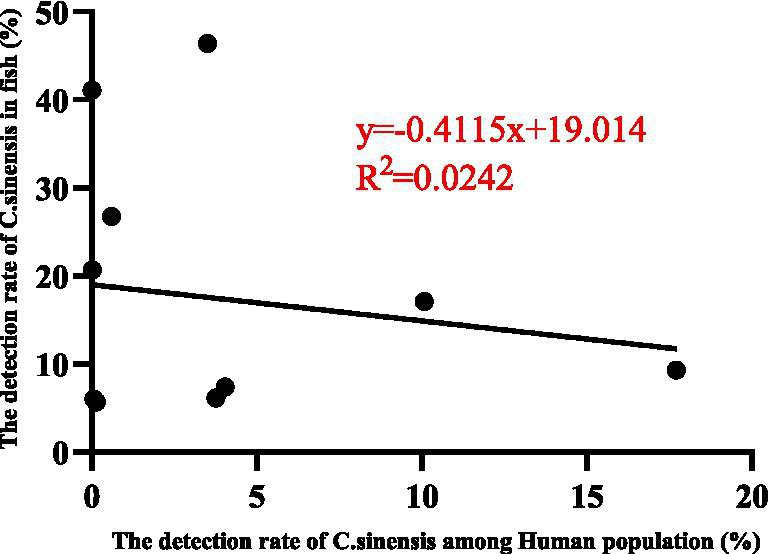
The concordance rate between the detection rates of *C. sinensis* in various second intermediate hosts and human populations across different provinces and regions (11 points) was analyzed utilizing the GraphPad PRISM version 9.0 software.

This review presents a comprehensive analysis of *C. sinensis* epidemiology across various host species in China, focusing on its prevalence in the secondary immediate hosts (freshwater fish species) and the definitive host (human populations). The aggregated detection rates indicate a prevalence of 15.71% (6,325/40,268) in freshwater species and 3.98% (60,306/1,513,994) in human populations. Notably, elevated prevalence rates of *C. sinensis* were observed among fish in the provinces of Heilongjiang, Liaoning, Shandong, Hubei, Sichuan, and Fujian. In contrast, high human infections were documented in the provinces of Jilin, Guangxi, Heilongjiang, Hunan, and Jiangxi. A weak correlation was identified between the prevalence of *C. sinensis* in freshwater fish and the human populations, as indicated by an R^2^ value of 0.0242 (Figure 6), indicating that additional factors beyond fish infection levels may play a significant role in influencing human infection rates.

The prevalence of *C. sinensis* across different fish species indicates that certain freshwater fish species exhibit a notably high rate of infection. These species include *P. parva*, *E. ilishaeformis*, *C. auratus*, and *A. nobilis*, many of which are regarded as optional ingredients in the preparation of “yusheng” in some regions of China. Thus, the consumption of “yusheng” may pose considerable public health risks in these areas where *C. sinensis* is epidemic (62).

The prevalence of *C. sinensis* among humans is influenced by several risk factors, including regions, sex, age, occupation, educational attainment, and ethnic groups (Table 3). Specifically, individuals residing in rural areas demonstrating a higher rate of infection in comparison to those living in urban towns and cities in China. Two reasons may contribute to this situation: Firstly, individuals residing in rural areas have a higher risk of contacting *C. sinensis*, primarily due to dietary practices such as the consumption of raw fish harboring this parasite. Secondly, the availability of medical resources and health education in rural regions is comparatively limited in China. Moreover, the prevalence of *C. sinensis* infection is elevated among individuals with high education levels. “Yusheng” represents a unique facet of culinary culture with high price in certain regions in China, including Guangxi and Guangdong Provinces (62). Individuals possessing advanced educational qualifications are observed to partake in “Yusheng” more frequently in urban towns and locales, despite having access to substantial medical resources and health education. In summary, these elderly male farmers, migrant workers, and medical personnel with higher levels of education are at an elevated risk of infection in epidemic areas. Consequently, it is imperative to implement targeted interventions aimed at modifying unhealthy dietary practices and enhancing food safety protocols to decrease infection rates within these at-risk populations. Additionally, ongoing monitoring and community engagement initiatives can further mitigate the transmission risks.

In recent years, Chinese government and researchers have made considerable efforts to investigate the epidemiological characteristics of *C. sinensis* infection across different host species within China. Nonetheless, several limitations in this body of research merit attention: (1) Snails, which are the primary intermediate hosts for *C. sinensis*, are frequently neglected in epidemiological studies conducted in China; (2) Likewise, domestic cats and dogs, which serve as definitive hosts for *C. sinensis* infection, are likely to interact with humans and indirectly increase their infection risk, have not been sufficiently studied regarding their prevalence in certain regions in China; (3) The enhanced Kato-Katz method has often been employed to assess the prevalence of *C. sinensis* across different definitive hosts; however, this method is known for its low sensitivity, which may result in false-negative outcomes in patients and other hosts with mild infections. Given these considerations, future research initiatives should focus on the development of novel diagnostic instruments and the improvement of epidemiological data related to *C. sinensis* infections in these under-explored host species.
