# Supplementary material for: Current knowledge on the prevalence and detection techniques of Clonorchis sinensis in China
Source: Front Vet Sci. 2025 Aug 13;12:1618633. doi: 10.3389/fvets.2025.1618633 (PMC12382394; doi:10.3389/fvets.2025.1618633)
Supplement: Supplementary file 1 [file Supplementary_file_1.docx]

**Supplementary Table 1**: The detail information of research that investigated the prevalence of *C. sinensis* in second intermediate hosts (mainly referred to freshwater fish) in China from 2010 to to April 30, 2025.

| **Title** | **First author** | **Language** | **Year of publication** | **Period of investigating** | **Province** | **Sample Size** | **No. of positive** | **Prevalence, %** | **Number** |
| --- | --- | --- | --- | --- | --- | --- | --- | --- | --- |
| Investigation of Clonorchis sinensis infection in the national surveillance site of Pucheng County, Fujian Province during 2016—2020 | Chen BJ | Chinese | 2021 | 2016-2020 | Fujian | 639 | 472 | 73.86 | 1 |
| Investigation on the parasitic infection of some marketed freshwater products in Fujian Province from 2014 to 2019 | Cai WW | Chinese | 2021 | 2014-2019 | Fujian | 2232 | 2 | 0.09 | 2 |
| Preliminary report and analysis on food borne parasites in Longyan City | Wu LH | Chinese | 2016 | No mentioned | Fujian | 43 | 1 | 2.33 | 3 |
| Investigation of the Epidemiological Characteristic of Clonorchiasis in Jiangmen Pengjiang District | Liu YH | Chinese | 2012 | No mentioned | Guangdong | 156 | 15 | 9.62 | 4 |
| Investigation on sanitation of freshwater aquaculture environments and Clonorchis sinensis intermediate host infection in a city of Pearl River Delta region,China | Wang M | Chinese | 2017 | 2016 | Guangdong | 437 | 19 | 4.35 | 5 |
| Monitoring results of food-borne parasite in commercially available food in Shenzhen City | Luo XR | Chinese | 2015 | 2006-2013 | Guangdong | 567 | 39 | 6.88 | 6 |
| The Current Status of Foodborne Parasitic Infections among the Population in Jiangmen and Countermeasures for Prevention and Control | Guan BY | Chinese | 2017 | No mentioned | Guangdong | 1120 | 67 | 5.98 | 7 |
| Epidemiological characterization of Clonorchis sinensis infection in humans and freshwater fsh in Guangxi, China | Xie YH | English | 2022 | 2016-2017 | Guangxi | 2098 | 203 | 9.68 | 8 |
| Investigation on the Infection Status of Clonorchis sinensis Cysticerci in Freshwater Fish in Linqiu New District, Guilin City | Li X | Chinese | 2018 | No mentioned | Guangxi | 199 | 11 | 5.53 | 9 |
| Investigation and analysis of the infection of Clonorchis sinensis metacercaria in freshwater fishes from Zhaoyuan County | Chang QC | Chinese | 2015 | No mentioned | Heilongjiang | 510 | 186 | 36.47 | 10 |
| Prevalence of Metacercariae of Clonorchis sinensis in Wild Freshwater Fishes from Nenjiang River around Qiqihaer City | Liu JX | Chinese | 2014 | 2013 | Heilongjiang | 1175 | 602 | 51.2 | 11 |
| Seasonal Analysis of Zoonotic Trematode Cysticercus Infections in Fish Sources | Zhang Y | Chinese | 2019 | 2017-2018 | Heilongjiang | 807 | 263 | 32.59 | 12 |
| Infection with Clonorchis sinensis (Cobbold, 1875) Metacercariae in Fish from the East Lake of Wuhan: Freshwater Fish in Urban Lakes May Act as Infection Sources of Liver Fluke | Jiang JN | English | 2024 | 2021-2022 | Hubei | 815 | 265 | 32.5 | 13 |
| Investigation of infection with Clonorchis sinensis in freshwater fish from Xianning City | Luo C | Chinese | 2011 | No mentioned | Hubei | 444 | 234 | 52.7 | 14 |
| Prevalence of fish-borne zoonotic trematode infection in Jilin Province, China | Wang YR | English | 2022 | 2020 | Jilin | 4122 | 931 | 22.59 | 15 |
| Epidemiological Status of Clonorchis sinensis Infection in Key Regions of Western Jilin Province in 2021 | Wang X | Chinese | 2023 | 2021 | Jilin | 1358 | 66 | 5.3 | 16 |
| Monitoring and analysis of food-borne parasites in freshwater products and meats in Jilin province | Shi B | Chinese | 2018 | 2015-2016 | Jilin | 312 | 11 | 3.53 | 17 |
| Prevalence of parasitic infections in fish from markets in Zhenjiang City from 2017 to 2020 | Li AH | Chinese | 2022 | 2017-2020 | Jiangsu | 1193 | 117 | 9.81 | 18 |
| Epidemiological investigation of second intermediate hosts of Clonorchis sinensis in Pizhou and Xinyi cities of Jiangsu Province | Jin XL | Chinese | 2015 | No mentioned | Jiangsu | 1117 | 251 | 22.47 | 19 |
| Survey of infectious status of metacercariae of Clonorchis sinensis in wild fresh water Pseudorasboea parva wuhu water | Jiang F | Chinese | 2018 | No mentioned | Jiangsu | 100 | 34 | 34 | 20 |
| The achievements of 3-year efforts of clonorchiasis control in the demonstration area of Xinfeng County in Jiangxi Province | Ge J | Chinese | 2020 | 2016 and 2018 | Jiangxi | 1872 | 145 | 7.75 | 21 |
| Survey on the Prevalence of Clonorchis sinensis Cysticercoids in Freshwater Fish and Shrimp Available in Shenyang City | Yang S | Chinese | 2012 | No mentioned | Liaoning | 178 | 85 | 47.75 | 22 |
| Prevalence and species identification of trematode metacercariae in Qiqihar, Northeast China | Zhang FY | English | 2024 | 2021 | Qinghai | 600 | 286 | 47.7 | 23 |
| Survey of infectious status of metacercariae of Clonorchis sinensis in wild freshwater fish of Liaocheng region of Shandong province | Liu CJ | Chinese | 2016 | 2014 | Shandong | 693 | 285 | 41.13 | 24 |
| Investigation on the animal foods contaminated with parasites in markets in Huangpu District of Shanghai from 2015 to 2017 | Lu L | Chinese | 2018 | 2015-2017 | Shanghai | 271 | 0 | 0 | 25 |
| An investigation on the status of wild freshwater fish and shrimp infected with metacercaria of clonorchis sinensis in Jinhua City | Yu XT | Chinese | 2015 | No mentioned | Zhejiang | 2326 | 131 | 5.63 | 26 |
| Survey on the Prevalence of Clonorchis sinensis Infection in Freshwater Fish in Tonglu County | Wu BY | Chinese | 2013 | No mentioned | Zhejiang | 248 | 14 | 5.65 | 27 |
| Current status and influencing factors of Clonorchis sinensis infection in rural areas of Ninghai County in Zhejiang Province | Gu MX | Chinese | 2017 | 2016 | Zhejiang | 381 | 51 | 13.39 | 28 |
| Prevalence of clonorchis sinensis infection in Western Area of Zhejiang Province | Chen HL | Chinese | 2013 | 2012 | Zhejiang | 2812 | 189 | 6.7 | 29 |

**Supplementary Table 2**: The detail information of research that investigated the prevalence of *C. sinensis* among human population in China from 2010 to April 30, 2025.

| **Title** | **First author** | **Language** | **Year of publication** | **Period of investigating** | **Province** | **Target** | **Sample Size** | **No. of positive** | **Prevalence, %** | **Number** |
| --- | --- | --- | --- | --- | --- | --- | --- | --- | --- | --- |
| Epidemic status of Clonorchis sinensis and soil?derived nematodes in Guangzhou，2010-2020 | Chen HY | Chinese | 2022 | 2010-2020 | Guangdong | Eggs | 41263 | 1291 | 3.13 | 1 |
| Analysis on surveillance results of clonorchiasis in Zhejiang Province from 2013 to 2022 | Chai YZ | Chinese | 2024 | 2013-2022 | Zhejiang | IgG | 1516 | 38 | 2.51 | 2 |
| Analysis on surveillance results of clonorchiasis in Zhejiang Province from 2013 to 2022 | Chai YZ | Chinese | 2024 | 2013-2022 | Zhejiang | Eggs | 52626 | 0 | 0 | 3 |
| Investigation on prevalence of soil-transmitted nematodes and Clonorchis sinensis in four counties of eastern Guangxi from 2015 to 2016 | Chen HS | Chinese | 2021 | 2015-2016 | Guangxi | Eggs | 3280 | 451 | 13.75 | 4 |
| Investigation and analysis on the prevalence of Clonorchis sinensis infection in Guizhou Province in 2015 | Li AM | Chinese | 2021 | 2015 | Guizhou | Eggs | 8838 | 37 | 0.42 | 5 |
| Investigation on the prevalence of Clonorchis sinensis infection in human population of Heilongjiang Province, 2015 | Tang L | Chinese | 2021 | 2015 | Heilongjiang | Eggs | 30280 | 747 | 2.47 | 6 |
| Surveillance of key parasitic diseases in populations of Baise City in Guangxi from 2016 to 2019 | Deng JG | Chinese | 2021 | 2016-2019 | Guangxi | Eggs | 14524 | 1464 | 10.08 | 7 |
| Surveillance on clonorchiasis in 2016—2019 in Heng county, Guangxi | Chen Y | Chinese | 2021 | 2016-2019 | Guangxi | Eggs | 3033 | 989 | 32.6 | 8 |
| Analysis on the prevalence of Clonorchis sinensis infection in Lingshan County, Guangxi from 2016 to 2019 | Huang GH | Chinese | 2021 | 2016-2019 | Guangxi | Eggs | 4363 | 212 | 4.86 | 9 |
| Investigation and analysis on intestinal parasitic infection in outpatient service of Guigang Center for Disease Control and Prevention from 2016 to 2019 | Li T | Chinese | 2022 | 2016-2019 | Guangxi | Eggs | 1406 | 417 | 29.66 | 10 |
| Analysis on the status of human infection in national surveillance sites for clonorchiasis in Xinfeng County during 2016-2019 | Yuan CH | Chinese | 2020 | 2016-2019 | Jiangxi | Eggs | 4034 | 300 | 7.44 | 11 |
| Epidemiological Analysis of Clonorchis sinensis Infection among Residents of Qitang Village, Xinfeng County (2016–2019) | Xiong YF | Chinese | 2020 | 2016-2019 | Jiangxi | Eggs | 788 | 116 | 14.7 | 12 |
| Investigation on the status of Clonorchis sinensis infection in population in Anhui Province from 2016 to 2020 | Cao GZ | Chinese | 2021 | 2016-2020 | Anhui | Eggs | 73702 | 27 | 0.04 | 13 |
| Surveillance and analysis of important human parasitic infections in Beijing during 2016—2020 | He ZY | Chinese | 2021 | 2016-2020 | Beijing | Eggs | 15200 | 0 | 0 | 14 |
| Investigation of Clonorchis sinensis infection in the national surveillance site of Pucheng County, Fujian Province during 2016—2020 | Chen BJ | Chinese | 2021 | 2016-2020 | Fujian | Eggs | 5553 | 26 | 0.47 | 15 |
| Data analysis of clonorchiasis surveillance in high endemic areas of Guangdong Province in 2016—2020 | Wang KY | Chinese | 2022 | 2016-2020 | Guangdong | Eggs | 5116 | 749 | 14.6 | 16 |
| Monitoring Outcomes of Major Human Parasitic Diseases and Investigation on Residents' Awareness and Behaviors in Tongzhou District, Beijing (2011–2022) | Chen LX | Chinese | 2024 | 2011-2022 | Beijing | Eggs | 1500 | 0 | 0 | 17 |
| A survey on the status of important human parasitic diseases in Beijing in 2015 | He ZY | Chinese | 2020 | 2015 | Beijing | Eggs | 1782 | 0 | 0 | 18 |
| Analysis on clonorchiasis surveillance in Guangxi Zhuang Autonomous Ｒegion from 2016 to 2020 | Lv GL | Chinese | 2021 | 2016-2020 | Guangxi | Eggs | 31671 | 2997 | 9.46 | 19 |
| Surveillance Outcomes and Analysis of Major Parasitic Diseases in Jinhu County (2016–2020) | Zhang CP | Chinese | 2021 | 2016-2020 | Jiangsu | Eggs | 5110 | 20 | 0.39 | 20 |
| Analysis of Surveillance Results for Key Human Parasitic Diseases in Jingxi City (2016–2020) | Wei B | Chinese | 2022 | 2016-2020 | Guangxi | Eggs | 5051 | 179 | 3.54 | 21 |
| Survey on infection statys of Clonorchis sinensis, Nanning city, 2016-2020 | Zeng XM | Chinese | 2022 | 2016-2020 | Guangxi | Eggs | 17255 | 5290 | 30.66 | 22 |
| Analysis on the surveillance results of clonorchiasis in Sichuan Province from 2016 to 2020 | Luo JW | Chinese | 2021 | 2016-2020 | Sichuan | Eggs | 143311 | 28 | 0.02 | 23 |
| Spatial?distribution?of?Clonorchiasis?in?Heilongjiang,?2016 - 2021? | Tang L | Chinese | 2023 | 2016-2021 | Heilongjiang | Eggs | 114905 | 4488 | 3.91 | 24 |
| Investigation of epidemic status of Clonorchiasis sinensis in Pucheng County from 2016 to 2021 | Yu WW | Chinese | 2024 | 2016-2021 | Fujian | Eggs | 6187 | 25 | 0.4 | 25 |
| Epidemiological and spatial distribution characteristics of Clonorchis sinensis human infections in Guangdong Province from 2016 to 2022 | Zhang GT | Chinese | 2024 | 2016-2022 | Guangdong | Eggs | 153188 | 5369 | 3.5 | 26 |
| Infection status of clonorchiasis sinensis and the knowledge, attitudes and practices towards the disease among human population in Xinfeng County of Jiangxi Province in 2016 and 2018 | Chen Z | Chinese | 2021 | 2016 and 2018 | Jiangxi | Eggs | 2216 | 366 | 16.52 | 27 |
| Investigation and analysis on the infection status of Clonorchis sinensis and its related knowledge-attitude-practice in population in Heyuan City from 2017 to 2020 | Qin LF | Chinese | 2021 | 2017-2020 | Guangdong | Eggs | 6430 | 330 | 5.13 | 28 |
| Epidemiological investigation and analysis of Clonorchis sinensis infection in a hospital in Panyu district，Guangzhou，from 2017 to 2021 | Liao LY | Chinese | 2023 | 2017-2021 | Guangdong | Eggs | 187166 | 6418 | 3.43 | 29 |
| A Study on the Seroprevalence of Clonorchis sinensis Antibodies Among Health Examination and Outpatient Patients at a Hospital in Chongzuo City, Guangxi (2018–2020) | Feng Y | Chinese | 2023 | 2018-2020 | Guangxi | IgG | 12735 | 3818 | 29.98 | **30** |
| Survey on Clonorchis sinensis infection and influencing factors, Jinhu county, 2018-2020 | Li SM | Chinese | 2022 | 2018-2020 | Jiangsu | Eggs | 3043 | 11 | 0.36 | 31 |
| Prevalence ?of ?clonorchiasis ?among ?permanent ?residents ?in ?Harbin municipality,?2018:?a?cross-sectional?survey | Zhang YM | Chinese | 2021 | 2018 | Heilongjiang | Eggs | 8980 | 351 | 3.91 | 32 |
| Analysis of the current status of Clonorchis sinensis infection in Xinfeng County, Jiangxi Province from 2020 to 2022 | Lan YM | Chinese | 2024 | 2020-2022 | Jiangxi | Eggs | 3142 | 202 | 6.43 | 33 |
| Survey and Analysis of Clonorchis sinensis Infection Among the Population of Guangxi in 2020 | Wei YL | Chinese | 2022 | 2020 | Guangxi | Eggs | 3243 | 145 | 4.47 | 34 |
| Analysis of Paragonimus hepatica infection in Heilongjiang Province in 2020 | Yuan S | Chinese | 2023 | 2020 | Heilongjiang | Eggs | 19194 | 331 | 1.72 | 35 |
| Surveillance and analysis of human intestinal helminth infection in Shandong Province in 2023 | Lv WX | Chinese | 2024 | 2023 | Shandong | Eggs | 14507 | 2 | 0.01 | 36 |
| Prevalence of intestinal helminth infections in Jiangsu Province, eastern China; a cross sectional survey conducted in 2015 | Dai Y | English | 2019 | 2015 | Jiangsu | Eggs | 12423 | 4 | 0.03 | 37 |
| Soil-transmitted helminths, intestinal protozoa and?Clonorchis sinensis infections in?southeast China | Feng Y | English | 2021 | 2014-2015 | Zhejiang | Eggs | 23552 | 1 | 0.004 | 38 |
| High prevalence of Clonorchis sinensis infection in Guangxi, Southern China | Jiang ZH | English | 2021 | 2019 | Guangxi | Eggs | 15683 | 1659 | 10.6 | 39 |
| Foodborne Parasites Dominate Current Parasitic Infections in Hunan Province, China | Liu XH | English | 2021 | 2016-2020 | Hunan | Eggs | 4428 | 70 | 1.58 | 40 |
| Interspecies Phylogenetic Analysis of Clonorchis sinensis in High-incidence Areas of Hunan Province, China | Long XL | English | 2021 | 2016-2020 | Hunan | Eggs | 188039 | 2595 | 1.38 | 41 |
| An investigation of Human Clonorchiasis prevalence in an Endemic County in Guangxi Zhuang Autonomous Region, China, 2016 | Xin HL | English | 2021 | 2016 | Guangxi | Eggs | 2282 | 1109 | 48.6 | 42 |
| Investigation of epidemic status of clonorchiasis sinensis in western regions of Jilin Province | Xu CX | Chinese | 2020 | 2017 | Jilin | Eggs | 4980 | 1220 | 24.5 | 43 |
| Current endemic status of Clonorchis sinensis infection in population of Jilin Province in 2015 | Wang X | Chinese | 2019 | 2015 | Jilin | Eggs | 24963 | 749 | 3 | 44 |
| Report on the National Survey of Key Human Parasitic Diseases in Songyuan City in 2017 | Chen XR | Chinese | 2018 | 2017 | Jilin | Eggs | 3159 | 468 | 14.8 | 45 |
| High endemicity of Clonorchis sinensis infection in Binyang County, southern China | Sun JL | English | 2020 | No mentioned | Guangxi | Eggs | 2521 | 728 | 0.289 | 46 |
| High prevalence of Clonorchis sinensis infections and coinfection with hepatitis virus in riverside villages in northeast China | Gao YH | English | 2020 | 2017 | Jilin | Eggs | 3068 | 899 | 29.3 | 47 |
| Survey on the Current Status of?Clonorchis sinensis?Infection in Key Areas of Western Jilin Province in 2021 | Wang X | Chinese | 2023 | 2021 | Jilin | Eggs | 5000 | 808 | 16.16 | 48 |
| Molecular Identification and Phylogenetic Analysis of Nuclear rDNA Sequences of Clonorchis sinensis Isolates From Human Fecal Samples in Heilongjiang Province, China | Zhang XL | English | 2019 | 2014-2015 | Heilongjiang | Nucleotide | 342 | 74 | 21.64 | 49 |
| Risk Factors for Clonorchis sinensis Infection in Residents of Binyang, Guangxi: A Cross-Sectional and Logistic Analysis Study | Xu M | English | 2021 | 2016-2017 | Guangxi | Eggs | 1977 | 405 | 20.49 | 50 |
| Investigation of the Epidemiological Characteristic of Clonorchiasis in Jiangmen Pengjiang District | Liu YH | Chinese | 2012 | 2011 | Guangdong | Eggs | 2501 | 537 | 24.47 | 51 |
| The current situation and prevention and control measures of foodborne parasitic infections among the population in Jiangmen area | Guan BY | Chinese | 2017 | 2011-2016 | Guangdong | Eggs | 4635 | 442 | 9.53 | 52 |
| Epidemiological and spatial distribution characteristics of Clonorchis sinen? sis human infections in Guangdong Province from 2016 to 2022 | Zhang GT | Chinese | 2024 | 2016-2022 | Guangdong | Eggs | 153188 | 5369 | 3.5 | 53 |
| Epidemiology and determinants of Clonorchis sinensis infection in Binyang County, Guangxi in 2022 | Zhu YY | Chinese | 2024 | 2022 | Guangxi | Eggs | 1007 | 156 | 15.49 | 54 |
| Study on the burden of clonorchiosis in a city of the pearl river delta from 2019 to 2023 | Zhong JF | Chinese | 2025 | 2019-2023 | Guangdong | Eggs | 5219 | 434 | 8.32 | 55 |
| Investigation and analysis of Clonorchis sinensis infection and related KAP status in population in Nanning City from 2021 to 2023 | Wei SL | Chinese | 2024 | 2021-2023 | Guangxi | Eggs | 8058 | 1534 | 19.04 | 56 |
| Detection of fecal parasites in patients in a hospital in Guangzhou | Li R | Chinese | 2024 | 2023 | Guangdong | Eggs | 24547 | 1018 | 4.15 | 57 |
| Investigation and Analysis of Igg Antibody Levels of Clonorchis Sinensis in Medical Examination Population in Nanning, Guangxi from 2020 to 2023 | He SQ | Chinese | 2024 | 2020-2023 | Guangxi | IgG | 10554 | 2976 | 28.2 | 58 |
| Clinical characteristics and pathogen spectra of parasitic infections in a tertiary hospital of Shanghai: A 13-year retrospective study | Zhang JM | English | 2022 | 2010-2022 | Shanghai | Eggs | 32545 | 136 | 0.42 | 59 |

**Supplementary Table 3**: The detail information of research that investigated the prevalence of *C. sinensis* among among other definitive animals (dogs, cats, and pigs).

| **Title** | **First author** | **Language** | **Year of publication** | **Period of investigating** | **Province** | **Target** | **Sample Size** | **No. of positive** | **Prevalence, %** | **Number** |
| --- | --- | --- | --- | --- | --- | --- | --- | --- | --- | --- |
| Prevalence of Clonorchis sinensis infection in dogs and cats in subtropical southern China | Lin | English | 2011 | 2006-2008 | Guangdong | Dogs | 503 | 103 | 20.5 | 1 |
| Prevalence of Clonorchis sinensis infection in dogs and cats in subtropical southern China | Lin | English | 2011 | 2006-2008 | Guangdong | Cats | 194 | 81 | 41.8 | 2 |
| Investigation of epidemic status of Clonorchiasis sinensis in Pucheng County from 2016 to 2021 | Yu | Chinese | 2024 | 2016-2021 | Fujian | Dogs and cats | 152 | 8 | 5.26 | 3 |
| Analysis on monitoring of liver-rot, Longmen county, 2017 | Xuan | Chinese | 2018 | 2017 | Guangdong | Dogs and cats | 27 | 0 | 0 | 4 |
| Investigation on the Prevalence of Zoonotic Parasites in Dogs and Cats in the Northeast Region and Analysis of Risk Factors | Qiu | Chinese | 2020 | No mentioned | Northeast China | Dogs | 255 | 1 | 0.39 | 5 |
| Preliminary Study on the Investigation of Host Infection by Abnormal Trematodes in Some Areas of Guangxi and Detection Methods | Huang | Chinese | 2014 | 2012-2013 | Guangxi | Dogs | 40 | 11 | 27.5 | 6 |
| Preliminary Study on the Investigation of Host Infection by Abnormal Trematodes in Some Areas of Guangxi and Detection Methods | Huang | Chinese | 2014 | 2012-2013 | Guangxi | Cats | 47 | 19 | 40.4 | 7 |
| Investigation and analysis of Clonorchis sinensis infection in cats and dogs sold in Quanzhou county, Guangxi | Tang | Chinese | 2024 | 2022-2023 | Guangxi | Dogs | 84 | 2 | 3.28 | 8 |
| Investigation and analysis of Clonorchis sinensis infection in cats and dogs sold in Quanzhou county, Guangxi | Tang | Chinese | 2024 | 2022-2023 | Guangxi | Cats | 107 | 66 | 61.68 | 9 |
| Investigation on the intestinal parasite infections in dogs and cats in Tangshan city | Sun | Chinese | 2016 | 2015 | Hebei | Cats | 85 | 7 | 8.25 | 10 |
| Investigation on the intestinal parasite infections in dogs and cats in Tangshan city | Sun | Chinese | 2016 | 2015 | Hebei | Dogs | 113 | 17 | 15.04 | 11 |
| Analysis on infection status of Clonorchis sinensis and other intestinal parasites in pet cats in Henan Province | Sui | Chinese | 2023 | 2021-2022 | Henan | Cats | 898 | 28 | 3.12 | 12 |
| Epidemiological survey on clonorchiasis and awareness of prevention and control knowledge in Xinfeng County of Jiangxi Province | Chen | Chinese | 2018 | 2016 | Jiangxi | Cats | 108 | 5 | 4.63 | 13 |
| Epidemiological survey on clonorchiasis and awareness of prevention and control knowledge in Xinfeng County of Jiangxi Province | Chen | Chinese | 2018 | 2016 | Jiangxi | Dogs | 226 | 3 | 1.33 | 14 |
| Epidemiological survey on clonorchiasis and awareness of prevention and control knowledge in Xinfeng County of Jiangxi Province | Chen | Chinese | 2018 | 2016 | Jiangxi | Pigs | 19 | 0 | 0 | 15 |
| Investigation on Clonorchis sinensis infections in marketed cats in Nanning city | Ou | Chinese | 2019 | 2016-2018 | Guangxi | Cats | 105 | 68 | 64.76 | 16 |
| Investigation on Clonorchis sinensis infection in dogs and cats in Nenjiang River basin Qiqihaer City | Sun | Chinese | 2015 | 2013-2014 | Liaoning | Cats | 15 | 1 | 6.67 | 17 |
| Investigation on Clonorchis sinensis infection in dogs and cats in Nenjiang River basin Qiqihaer City | Sun | Chinese | 2015 | 2013-2014 | Liaoning | Dogs | 35 | 3 | 8.57 | 18 |
| Establishment of detection methods and preparation of subunit vaccines of clonorchiasis sinensis | Duan | Chinese | 2024 | No mentioned | Jilin | Dogs | 63 | 8 | 12.7 | 19 |
| Investigation of the rate of intestinal parasite infection in dogs and cats in the City of Tangshan | Liu | Chinese | 2011 | 2010 | Hebei | Dogs and cats | 100 | 3 | 3 | 20 |
| Investigation on the Infection of Clonorchis sinensis in Dogs in Fuxing Town, Wangqing County | Gao | Chinese | 2021 | No mentioned | Jilin | Dogs | 83 | 28 | 33.73 | 21 |
| Survey on Clonorchis sinensis infection and influencing factors, Jilin county,2018-2020 | Li | Chinese | 2022 | 2018-2020 | Jiangsu | Cats | 62 | 2 | 3.23 | 22 |
| Survey on Clonorchis sinensis infection and influencing factors, Jilin county,2018-2020 | Li | Chinese | 2022 | 2018-2020 | Jiangsu | Dogs | 61 | 1 | 1.64 | 23 |
| Survey on Clonorchis sinensis infection and influencing factors, Jilin county,2018-2020 | Li | Chinese | 2022 | 2018-2020 | Jiangsu | Pigs | 60 | 0 | 0 | 24 |
